# Pro-tumoral immune cell alterations in wild type and *Shb*-deficient mice in response to 4T1 breast carcinomas

**DOI:** 10.18632/oncotarget.24643

**Published:** 2018-04-10

**Authors:** Xiujuan Li, Kailash Singh, Zhengkang Luo, Mariela Mejia-Cordova, Maria Jamalpour, Björn Lindahl, Ganlin Zhang, Stellan Sandler, Michael Welsh

**Affiliations:** ^1^ Department of Medical Cell Biology, Uppsala University, Uppsala 75123, Sweden; ^2^ Department of Medical Biochemistry and Microbiology, Uppsala University, Uppsala 75123, Sweden; ^3^ Cyrus Tang Hematology Center, Collaborative Innovation Center of Hematology, State Key Laboratory of Radiation Medicine and Protection, Soochow University, Suzhou 215123, China

**Keywords:** breast cancer, metastasis, angiogenesis, innate immunity, regulatory T cells

## Abstract

To assess mechanisms responsible for breast carcinoma metastasis, 4T1 breast carcinomas were grown orthotopically in wild type or *Shb* knockout mice. Tumor growth, metastasis, vascular characteristics and immune cell properties were analyzed. Absence of *Shb* did not affect tumor growth although it increased lung metastasis. *Shb* knockout mouse tumors showed decreased redness and less developed vascular plexa located at the periphery of the tumors. No difference in overall tumor vascular density, leakage or pericyte coverage was noted between the genotypes although the average vessel size was smaller in the knockout. Tumors induced an increase of CD11b^+^ cells in spleen, lymph node, thymus, bone marrow and blood. Numbers of *Shb* knockout CD11b/CD8^+^ cells were decreased in lymph nodes and bone marrow of tumor bearing mice. Mice with tumors had reduced numbers of CD4^+^ lymphocytes in blood/lymphoid organs, whereas in most of these locations the proportion of CD4^+^ cells co-expressing FoxP3 was increased, suggesting a relative increase in Treg cells. This finding was reinforced by increased blood interleukin-35 (IL-35) in wild type tumor bearing mice. *Shb* knockout blood showed in addition an increased proportion of IL-35 expressing Treg cells, supporting the notion that absence of *Shb* further promotes tumor evasion from immune cell recognition. This could explain the increased number of lung metastases observed under these conditions. In conclusion, 4T1 tumors alter immune cell responses that promote tumor expansion, metastasis and escape from T cell recognition in an *Shb* dependent manner.

## INTRODUCTION

Metastasis is usually the ultimate cause of cancer-induced death in patients afflicted by breast carcinoma. This is a complicated multi-step process, involving epithelial-mesodermal transition (EMT), vascular and lymphatic leakage, decreased pericyte coverage, promotion of tumor cell expansion by stromal cells such as tumor-associated macrophages, escape from immune surveillance mechanisms and the ability to seed at distant locations [1]. Tumor angiogenesis has received a considerable amount of attention in conjunction with breast cancer growth and metastasis [2, 3]. Although treatment with angiogenesis inhibitors improves progression-free survival, these lack an effect on overall survival [4]. Tumor-associated macrophages may also promote tumor growth and metastasis, either performing a supporting role as stromal cells providing a microenvironment favorable for tumor proliferation [5, 6] or by suppressing the immune response against tumor cells as myeloid-derived suppressor cells (MDSC) [7]. Recently, escape from immune surveillance has become evident as a mechanism operating in order for tumors to evade a devastating immune attack [8, 9]. Specifically concerning breast cancer, several reports indicate that immune suppressing regulatory T (Treg) cells become activated and prevalent in both blood and the tumor [10–12]. Treg cells produce anti-inflammatory cytokines such as interleukin-35 [13–15] that can block the proliferation of both CD4^+^ and CD8^+^ T cells and T helper 1 (Th1) and Th17 cells [16, 17]. Furthermore, it has been reported that CD8^+^ T cells play a major anti-tumoral role in breast carcinomas, further emphasizing the relevance of Tregs in this scenario [18]. Moreover, an increase of circulating IL-35 has been demonstrated in patients with breast cancer compared to healthy controls [19].

Src homology-2 domain protein B (SHB) is a ubiquitously expressed multi-domain adapter protein that assembles signaling complexes, which convey signals downstream of receptors such as vascular-endothelial growth factor (VEGF) receptor 2 or the T cell receptor [20, 21]. The *Shb* knockout mouse displays phenotypes related to reproduction [22, 23], glucose homeostasis [24, 25], the vasculature [26, 27], hematopoiesis [28] and atopic dermatitis [29, 30]. In tumor biology, absence of SHB aggravates *BCR-ABL* induced myeloid leukemia [31], whereas solid tumor growth is reduced due to impaired angiogenesis [26, 32]. The *Shb* knockout solid tumor phenotype displays inflammatory characteristics [21, 32, 33] and this has consequences for B16F10 melanoma metastasis, which was increased in *Shb* deficient hosts [33].

Considering the tremendous clinical importance of understanding basic mechanisms responsible for metastasis, we decided to investigate breast cancer 4T1 tumor growth and metastasis in relation to *Shb* deficiency by assessing tumor vasculature, innate immunity and adaptive immunity. We observe that 4T1 tumors cause major changes in myeloid and T cell populations that would be predicted to support tumor growth and metastasis. These effects were in some instances augmented by the absence of SHB, providing a likely explanation for increased lung metastasis.

## RESULTS

### Characteristics of 4T1 tumor bearing mice

**Figure 1:**
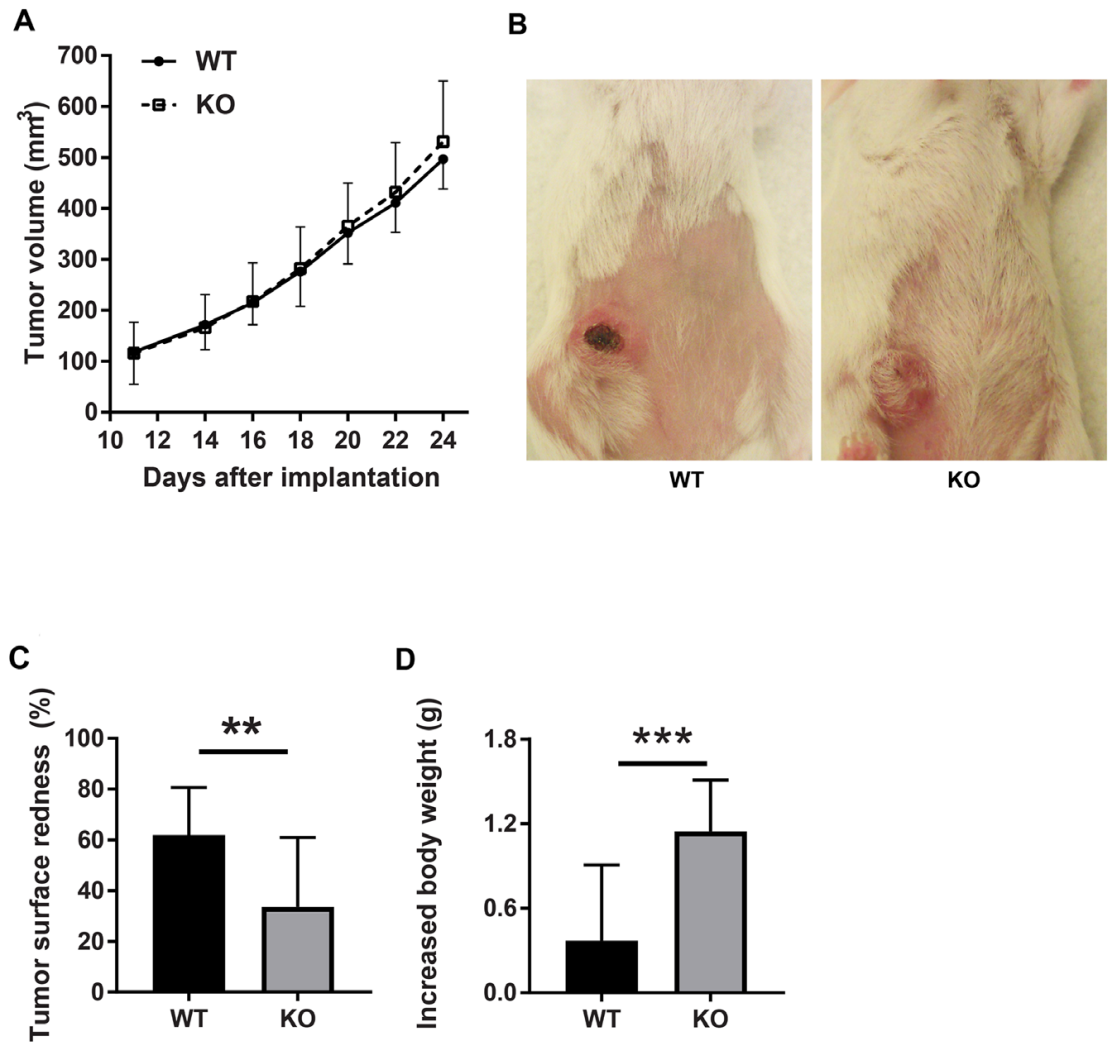
Tumor characteristics **(A)** Tumor growth curve. **(B)** Tumor redness. **(C)** Quantification of tumor redness as percent of tumor surface. **(D)** Increased mouse body weight. Breast carcinoma 4T1 cells were injected orthotopically into wild type or *Shb* knockout Balc/c mice. Tumor growth was monitored using a caliper. Tumor redness was estimated visually. Means ± SD are given. ^**^ and ^***^ indicate p< 0.01 and 0.001, respectively by Students’ t-test. N=23 mice each genotype.

Tumor growth was slightly increased in the absence of *Shb* although the effect failed to reach statistical significance (Figure 1A). Visual inspection revealed red tumors in wild type mice, unlike the tumors grown on the *Shb* deficient background (Figure 1B-1C). Hemorrhages or blood filled areas are frequently observed in 4T1 tumors [34] and apparently these may cause overlying scabs as seen in the figure. The decreased redness is reminiscent of what was observed in RIP-Tag2 insulinomas [32], which was interpreted to suggest a more inflammatory than angiogenic tumor phenotype as a consequence of *Shb*-deficiency [21]. Tumor bearing mice of both genotypes exhibited splenomegaly (0.67 ± 0.07 g for knockout and 0.67 ± 0.05 g for wild type) and weight gain, of which the latter was significantly larger in *Shb* knockout mice (Figure 1D).

**Figure 2:**
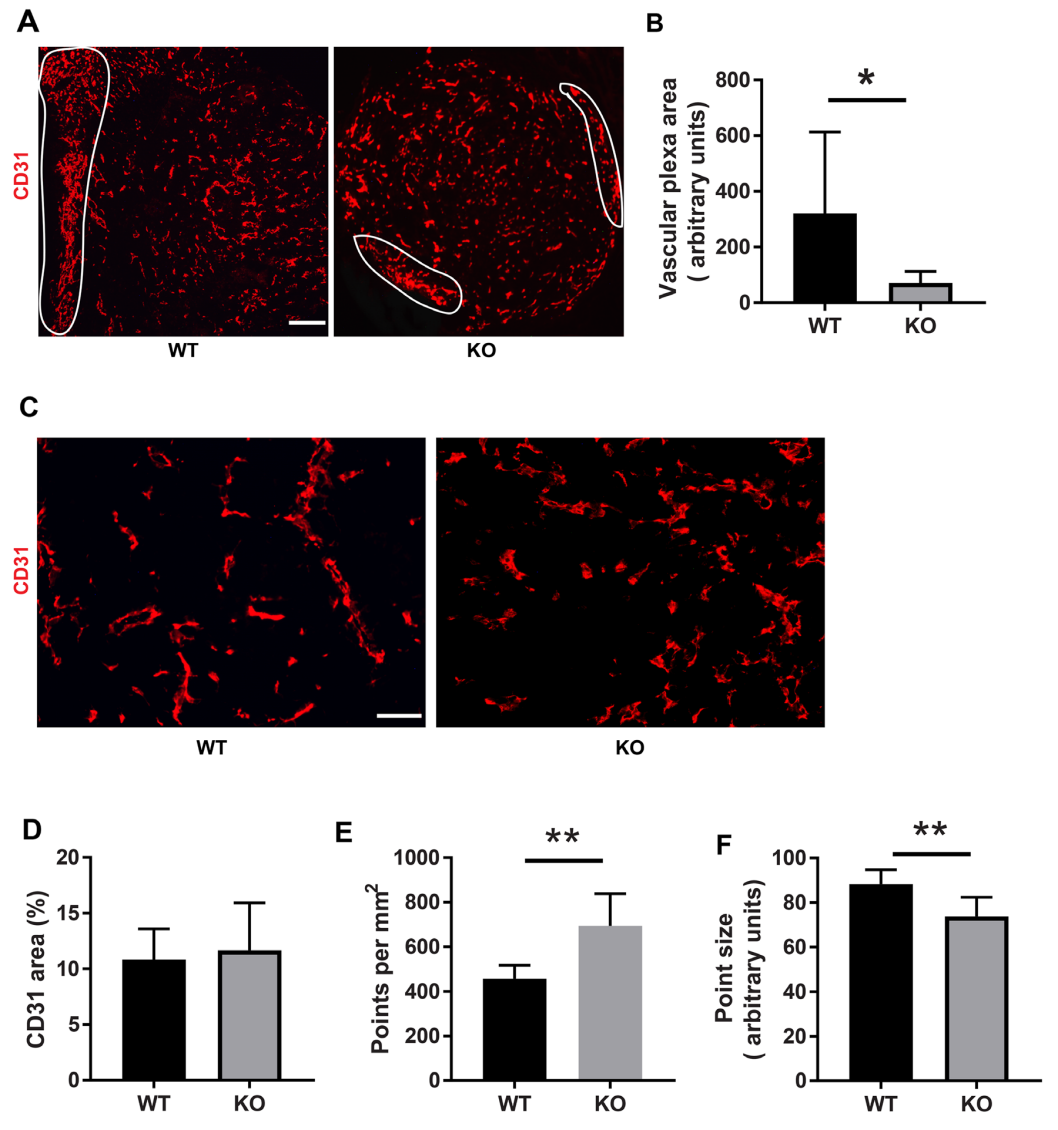
Tumor vasculature **(A)** CD31 staining of tumors with peripheral vascular plexa indicated at low magnification (50x, scale bar 200 μm). **(B)** Quantification of peripheral vascular plexus area. **(C)** Higher magnification (200x, scale bar 50 μm) images of vasculature inside the tumor. **(D)** Quantification of vascular density as percent area. **(E)** Quantification of number of vessels per image. **(F)** Quantification of average vessel size (point size). Tumors of both genotypes were stained for CD31 and Hoechst. Peripheral vascular plexa were marked as shown and their percentage area were calculated by ImageJ. ImageJ was also used to determine the percent CD31 positive area as well as average point size and average point number. Means ± SD are given. ^*^ and ^**^ indicate p<0.05 and p<0.01, respectively, by Students’ t-test. N= 6 mice each genotype.

**Figure 3:**
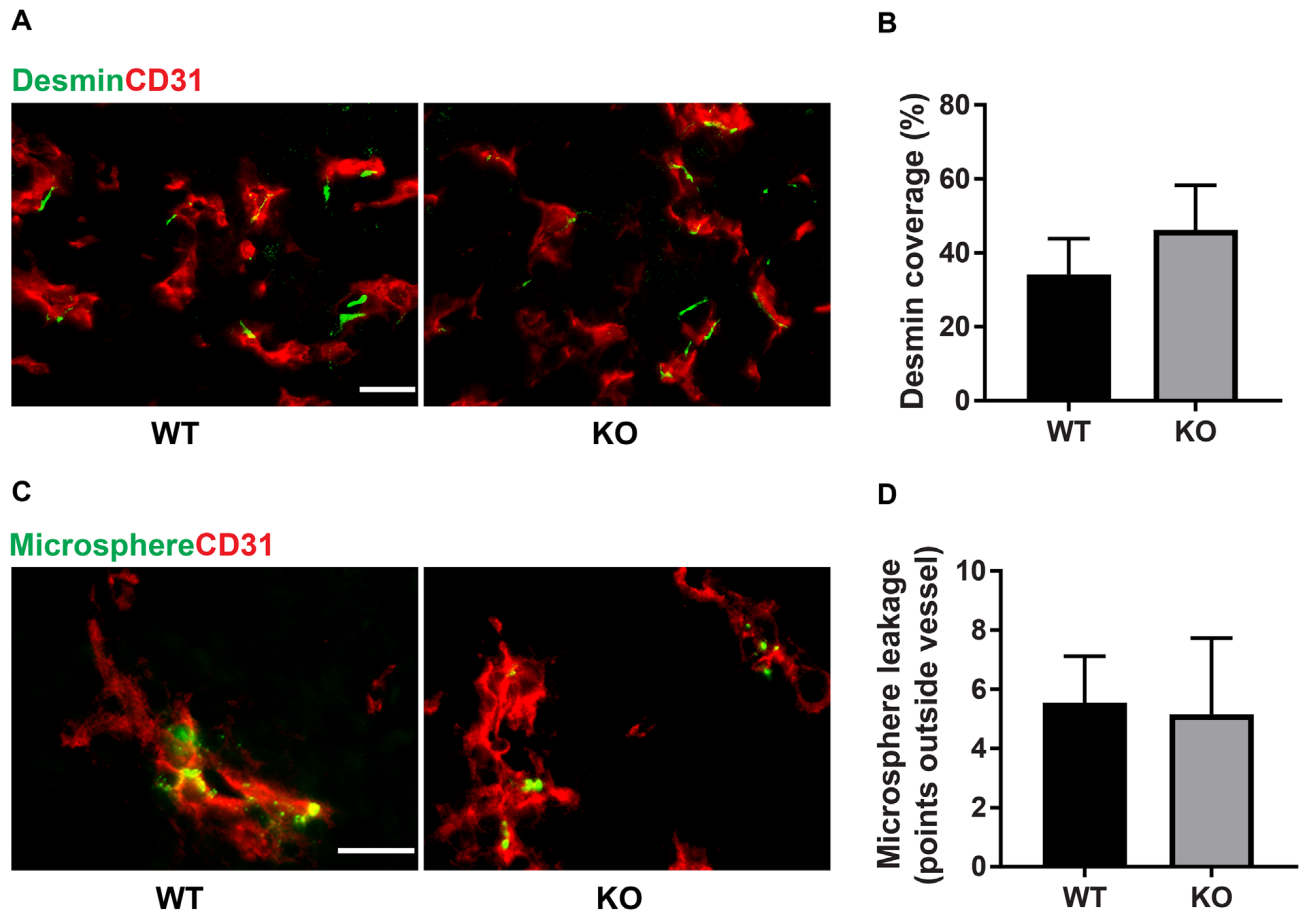
Pericyte coverage and vascular leakage **(A)** Staining for CD31 and desmin. Scale bar 20 μm. **(B)** Quantification of pericyte coverage. The length of desmin-positive staining (juxtaposed to CD31 positive staining) was measured and divided by the total vessel circumference for each vascular structure. **(C)** Extravasated microspheres representing vascular leakage and CD31 staining. Scale bar 20 μm. **(D)** Extravasated microspheres were counted and the graph shows these numbers for each genotype. Means ± SD are given. N= 4 mice each genotype.

**Figure 4:**
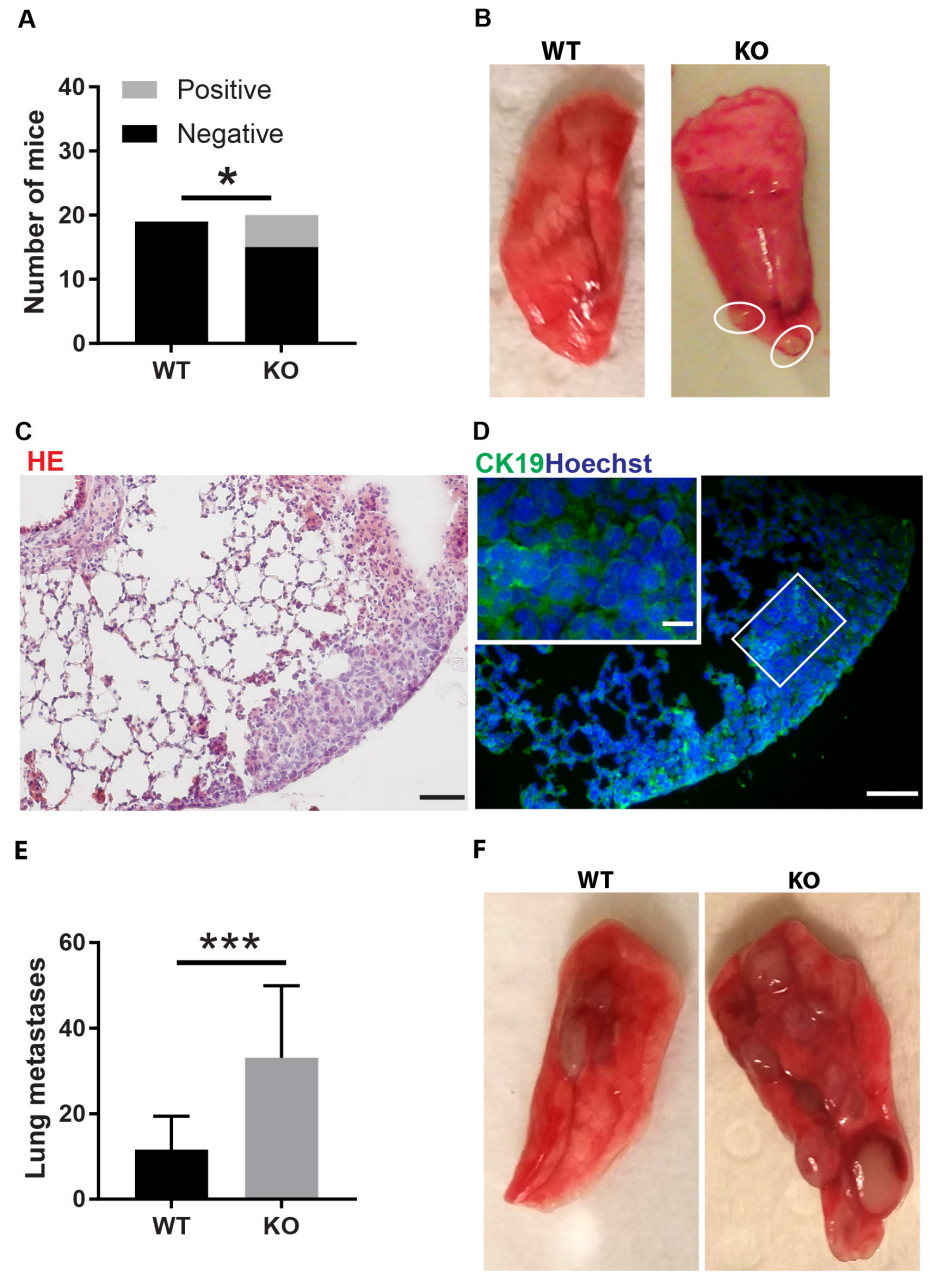
Tumor lung metastasis **(A)** Quantification of mice with and without metastases. **(B)** Picture of wild type lung lobe without metastases and *Shb* knockout lung lobe with two metastases. **(C)** HE staining of lung with metastases. Scale bar 50 μm. **(D)** Staining with the CK19 mammary epithelial marker. Scale bars 50 and 20 μm. In A-D, lung metastases were scored at 25 days when the primary tumors reached a critical size close to 1 cm^3^ and organs were collected for further analysis after sacrifice of the mice. **(E)** Lung metastasis per mouse after resection of primary tumor followed by an additional 8-14 days. **(F)** Picture of representative wild type and *Shb* knockout lung lobes after primary tumor resection with two metastases visible in wild type and eight metastases visible in *Shb* knockout. Means ± SD are given. N = 19-20 mice in (A–D) ^*^indicates p<0.05. N = 14-16 in (E–F) ^***^indicates p<0.001.

The difference in tumor color prompted us to investigate the tumor vasculature. Tumors grown on wild type mice exhibited prominent vascular plexa at the periphery of the tumors (Figure 2A) and these were significantly more pronounced compared with tumors grown on *Shb* knockout mice (Figure 2B), which probably explains the red appearance of the wild type tumors (Figure 1B). Inside the tumor, there was no difference in vascular density between the genotypes, although the tumors grown on knockout mice had more but smaller vessels (Figure 2C-2F), suggesting that different angiogenic cues were operating under these conditions. There was no difference in vascular leakage or pericyte coverage between the genotypes (Figure 3). Infiltration of CD8^+^, CD4^+^ and CD68^+^ cells was readily detectable in the tumors regardless of host genotype (Supplementary Figure 1). Lung metastasis was significantly increased in *Shb* deficient mice when the primary tumor reached a critical size of less than 1 cm^3^ at day 25 after cell injection and the mouse was sacrificed for further analysis (Figure 4A-4D). Metastasis was similarly increased when mice were subject to primary tumor resection at that time followed by an additional 8-14 days (Figure 4E-4F). Seeding of lung metastases after tail vein injections (Supplementary Figure 2) was not affected by the *Shb* knockout genotype, suggesting that expansion of the primary tumor was essential for increased metastasis occurring in the absence of *Shb*.

### Tumor gene expression

Total tumor expression of various genes potentially relevant for angiogenesis and immune responses was assessed by RT-qPCR. None of the genes tested (*Vegfa, Vegfc, Kdr* (VEGFR2), *Cdh5*, *Itgam* (CD11b), *Cd4, Cd8a, Foxp3, Angpt2* (Angiopoietin-2)*, Cxcl12, Ctla4, Cd274* (Pdl1), *Ifng*) showed any significant difference in expression between the genotypes (Supplementary Figure 3).

### Tumor induced alterations of myeloid cell composition

**Figure 5:**
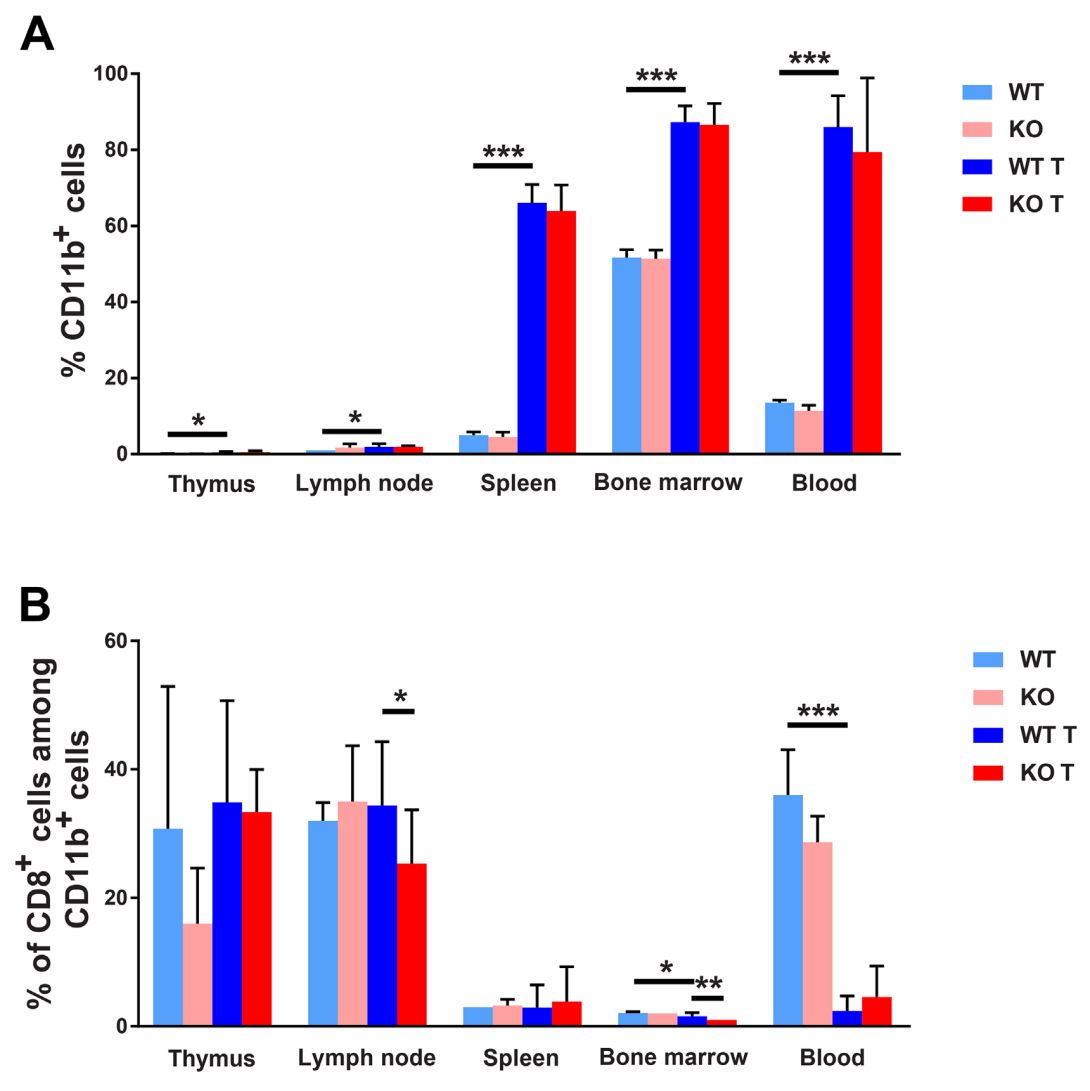
FACS staining for myeloid cell markers **(A)** CD11b positive cells in different lymphoid organs. **(B)** CD8/CD11b positive cells are given in percent of CD11b positive cells. Lymphoid organs were collected and dispersed at the time of sacrifice of non-tumor or tumor bearing mice, *ie* on day 25 after injection of the tumor cells or at the same time of age matched controls. Cells were stained for CD11b and CD8 and subjected to FACS analysis. Means ± SD are given. ^*^, ^**^ and ^***^ indicate p<0.05, p<0.01 and p<0.001, respectively, by Fisher’s LSD test comparing with wild type tumor group. N= 4 for non-tumor and 7-10 for tumor mice.

Different hematopoietic/lymphoid organs were subject to flow cytometric analysis by fluorescence activated cell sorting (FACS) in order to understand tumor-induced immune responses. All tissues (thymus, lymph nodes, spleen, bone marrow and blood) exhibited increased percentages of CD11b (*Itgam*) expressing cells in response to 4T1 tumor growth (Figure 5A). The effect in relative terms was quite prominent in thymi and spleens, especially when considering the tumor-induced increase in spleen size that simultaneously occurred. The increase was equally large in both genotypes. Considering CD11c (*Itgax*) dendritic cells, the tumor effect on its presence in lymphoid organs was less consistent. In spleens and bone marrows, the proportions of CD11c^+^ cells were decreased whereas this cell population was increased in lymph nodes (Supplementary Figure 4A). Tumors contained about 10% CD11b^+^ cells and there was no effect of genotype on their abundance (Supplementary Figure 5). In summary, tumors provoke a major increase of CD11b^+^ myeloid cells in lymphoid tissues and this effect may play a role in promoting tumor expansion. FACS plots showing the gating strategy can be seen in Supplementary Figure 6.

### Tumor induced alterations of CD11b/CD8^+^ and CD11c/CD8^+^ cell composition

Expression of CD11b on recently activated CD8^+^ effector cells has been described and related to anti-viral responses [35]. We thus decided to investigate the presence of this cell type in response to 4T1 tumors on wild type and *Shb* knockout backgrounds (Figure 5B). In lymph nodes and bone marrow there was a slight reduction of this cell type when calculated as percentages of CD11b^+^ cells in *Shb* knockout tumor-bearing mice compared with tumor-bearing wild type mice. On the other hand, tumors strongly reduced CD8^+^ CD11b^+^ cells in blood. The CD11b/CD8 double-positive cell population was also calculated in percent of the total cell population and was found to be slightly increased in lymph nodes from wild type tumor-bearing mice compared with wild type non-tumor mice and tumor-bearing *Shb* knockout mice (Supplementary Figure 7). *Shb* deficiency reduced this cell population among bone marrows in tumor–bearing mice. In summary, the data support the notion that in total there is a less active anti-tumor CD8^+^ T cell response in *Shb* deficiency.

CD11c/CD8^+^ cells have been reported to participate in ovalbumin and tumor induced cell mediated immunity [36, 37]. We therefore investigated the presence of this cell population in response to 4T1 tumors (Supplementary Figure 4B) when expressed in percent of CD11c^+^ cells. Tumors increased the proportion of this cell population in thymus, spleen and bone marrow whereas it was reduced in lymph nodes of *Shb* knockout mice with tumors when calculated as a percentage of the parent population. When CD11c/CD8 double positive cells were calculated in percent of the total cell population, this cell type was decreased in spleen, bone marrow and blood whereas it was increased in lymph nodes (Supplementary Figure 7). Thus, these findings further support an overall suppression of anti-tumor responses in tumor bearing mice.

### Tumor induced alterations of Treg cell composition

**Figure 6:**
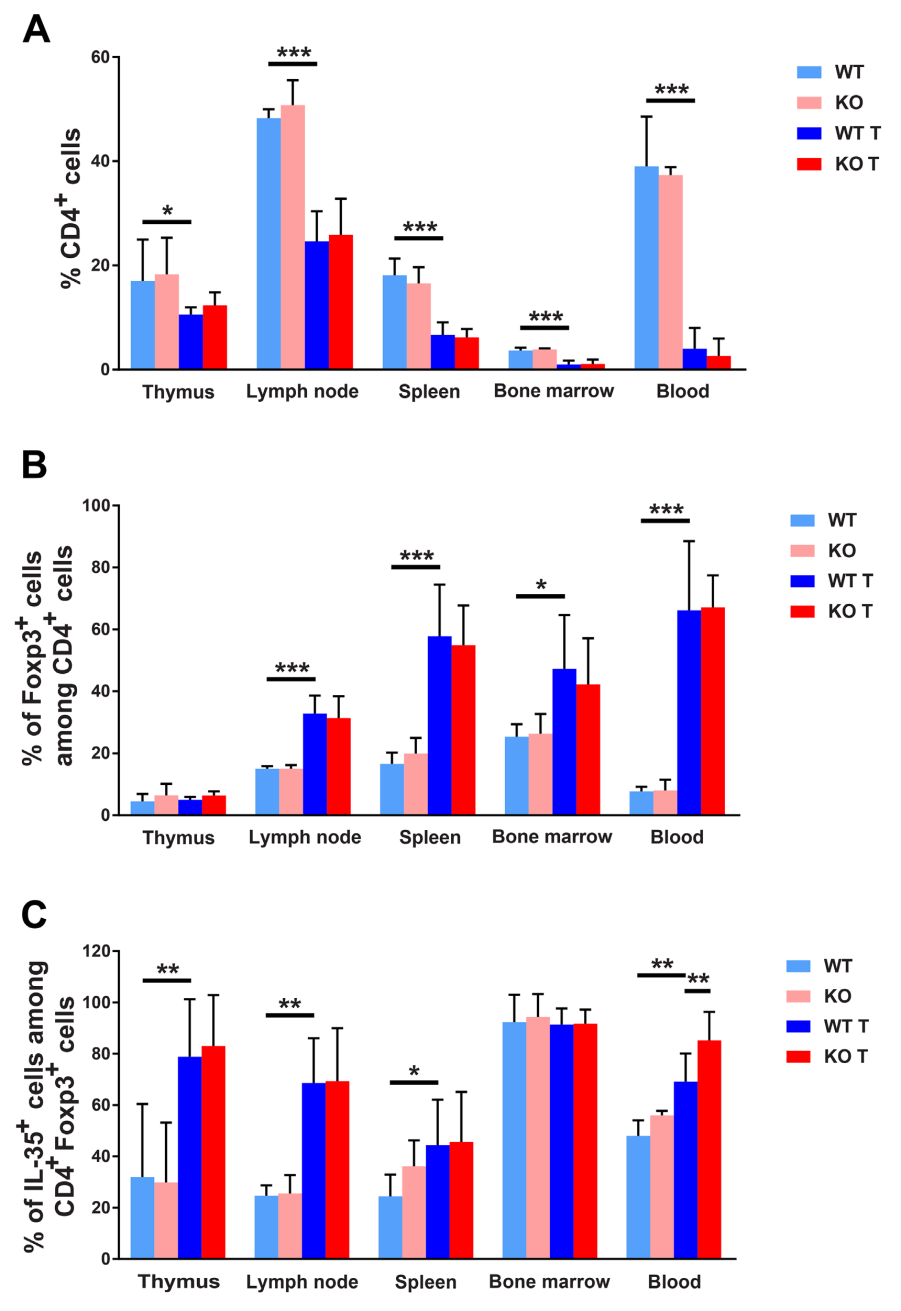
FACS staining for CD4+ Tregs **(A)** CD4 positive cells (percent) in different lymphoid organs. **(B)** Foxp3 positive cells as percentage of CD4+ cells. **(C)** IL-35 positive cells in percent of FoxP3/CD4 positive cells. Organs were collected at the time of sacrifice of non-tumor or tumor bearing mice, single cell suspensions prepared and stained for CD4, FoxP3, Ebi3 and IL-12a. Means ± SD are given. ^*^, ^**^ and ^***^ indicate p<=0.05, 0.01 and 0.001, respectively when compared as indicated with wild type tumor mice using Fisher’s LSD test. N= 4 for non-tumor and 7-10 for tumor mice.

Recent studies have implicated the importance of tumor evasion from immune attack and one mechanism by which this may occur is via activation of IL-35 producing Treg cells [8]. Consequently, cells from thymi, lymph nodes, spleens, bone marrows, blood and tumors were stained for the Treg markers FoxP3 and IL-35. All the tissues examined showed lower percentages of CD4+ cells in tumor bearing mice (Figure 6A). However, in lymph nodes, spleens, bone marrows and blood there was a relative increase in the fraction CD4/FoxP3^+^ cells (Figure 6B) and furthermore, thymi, lymph nodes, spleens and blood displayed elevated percentages of CD4/FoxP3/IL-35 expressing cells (Figure 6C). The latter population was additionally increased in blood from *Shb* knockout mice suggesting a selective preference for expansion of Treg cells in the absence of SHB protein.

In lymphoid organs, mixed responses to tumors were noted with regard to CD8^+^ cell populations, showing decreased numbers of this cell type in spleens, bone marrows and blood and increased numbers in thymi (Supplementary Figure 8A). The percentages CD8/FoxP3+ and CD8/FoxP3/IL-35+ cells (Supplementary Figure 8B-8C) were not altered in a consistent pattern. The percentage of CD8/FoxP3^+^ cells was decreased in lymph nodes and increased in blood whereas the CD8/FoxP3/IL-35 cell percentages were increased in lymph nodes and thymi and slightly decreased in bone marrows, suggesting that the effects on CD8^+^ Treg cells were modest in response to 4T1 breast cancers. No detectable differences in the tumoral CD4^+^ cell populations were noted (Supplementary Figure 5). CD8^+^ cells could not be detected in tumor samples due to high tumor cell autofluorescence (results not shown). The findings thus suggest that 4T1 tumors induce a CD4^+^ Treg response that is additionally augmented by absence of *Shb* and that this effect promotes tumor expansion and metastasis.

### Tumor induced plasma IL-35 concentrations

**Figure 7:**
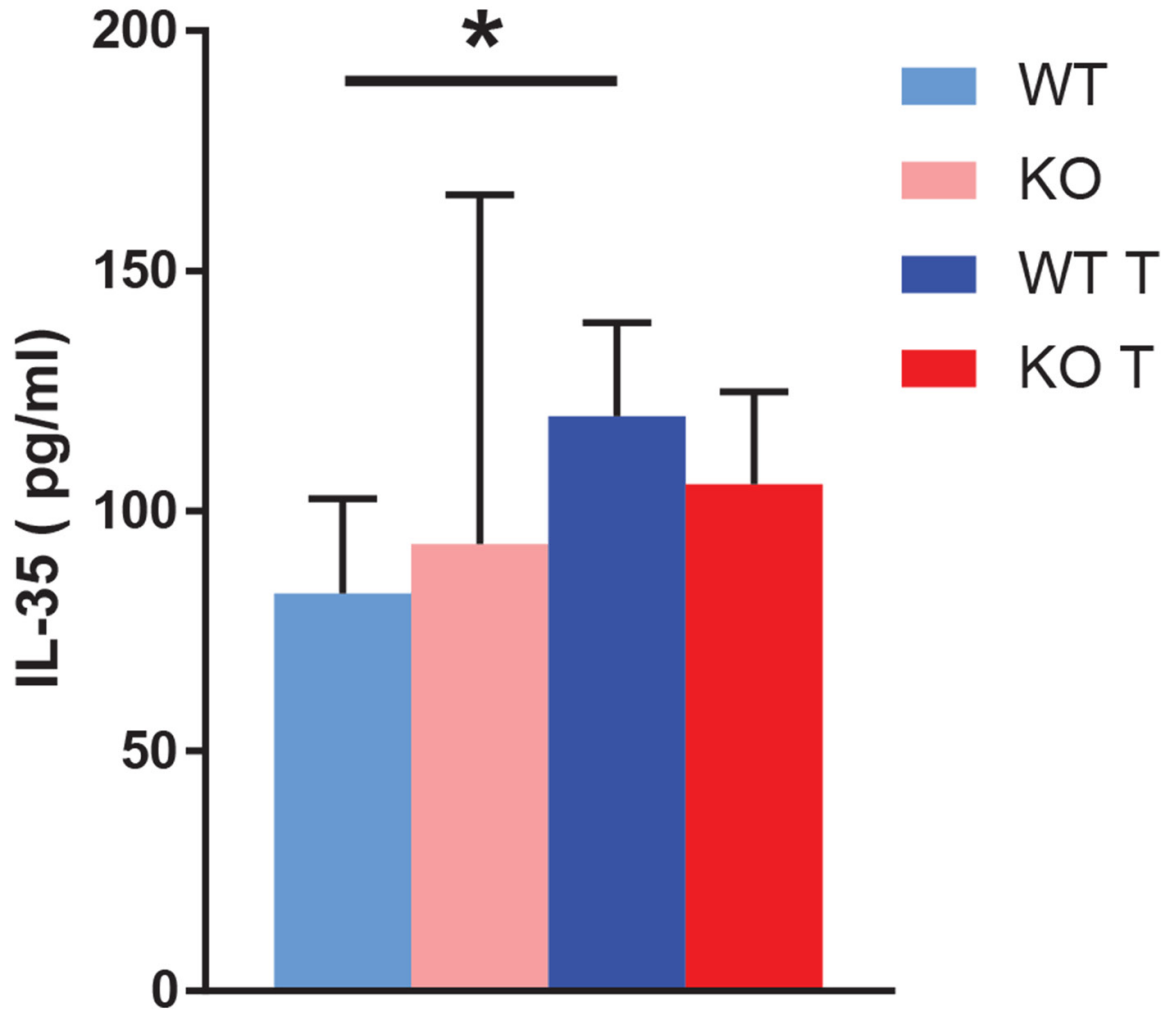
Blood IL-35 content Blood IL-35 was determined by ELISA at the time of sacrifice of non-tumor and tumor bearing mice. Means ± SD are given. ^*^ indicates p<0.05 when compared with wild type non-tumor. N= 3 for non-tumor and 7 for tumor mice.

Tumors increased the blood IL-35 concentration in wild type mice (Figure 7), further supporting the notion that an IL-35^+^ Treg cell response participates in tumor immune cell evasion. These data are in agreement with findings in humans showing that circulating IL-35 levels are increased in patients with breast cancer compared to healthy controls [19]. No difference was observed in knockout mice.

### Spleen and lymph node gene expression

**Figure 8:**
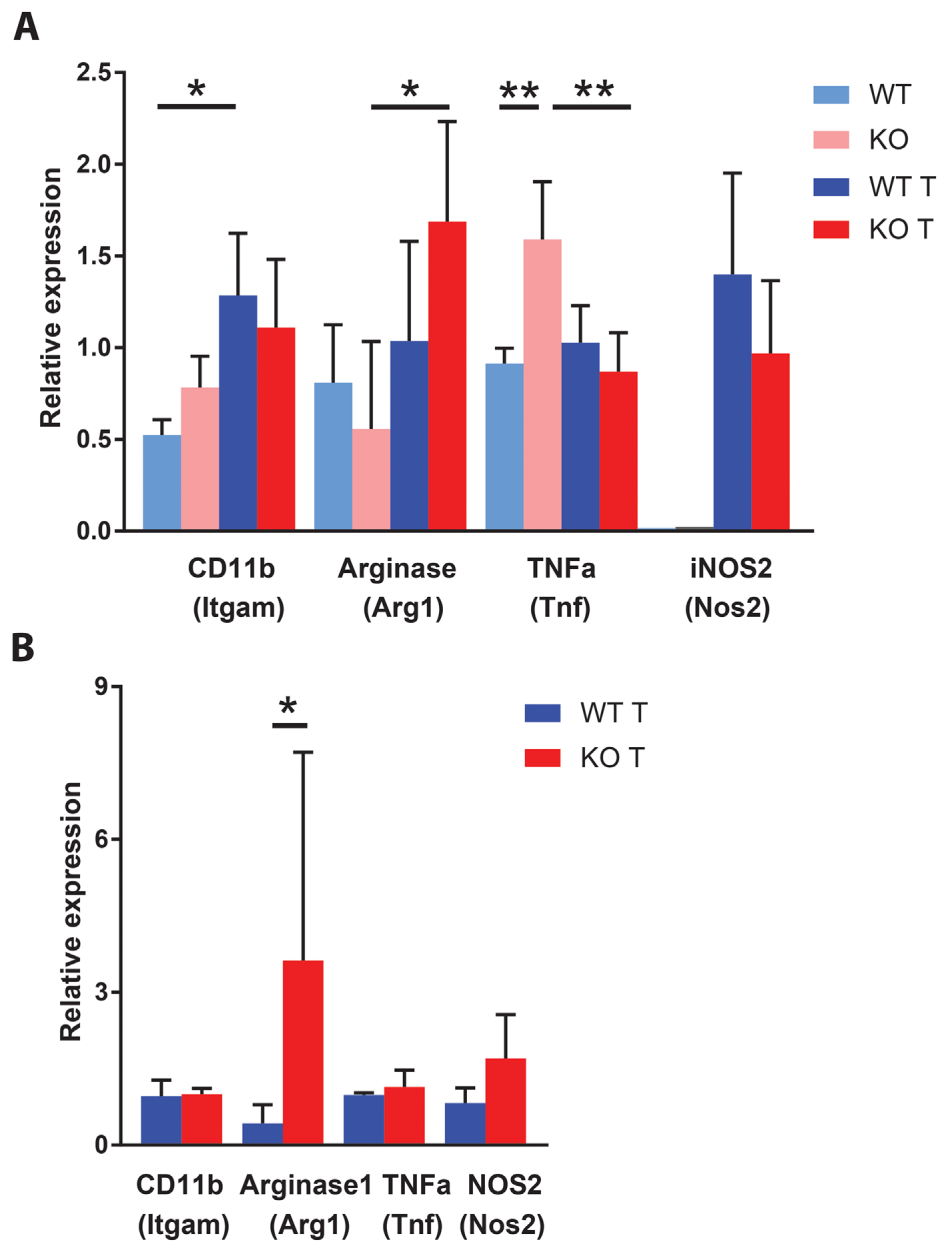
Expression of macrophage M1 (*Tnf* and *Nos2*) and M2 (Arg1) markers in spleen and lymph nodes **(A)** Spleen CD11b positive cells were isolated for RNA preparation at the time of sacrifice of non-tumor and tumor mice. The smaller amount of RNA obtained from non-tumor spleen CD11b positive cells precluded determination of *Nos2* gene expression. N= 3 for non-tumor and 6-7 for tumor. **(B)** Gene expression of CD11b positive cells from inguinal lymph nodes of tumor bearing mice at the time of sacrifice. N= 3 for *Itgam*, *Tnf* and *Nos2* and N=9-10 for *Arg1.* Means ± SD are given. Lymph nodes were too small in non-tumor bearing mice for preparing sufficient amounts of RNA. ^*^ and ^**^ indicate p<0.05 and p<0.01, respectively.

Myeloid cell expression of M2 markers has been proposed to facilitate tumor expansion [6, 38]. We decided to investigate the expression of genes related to the M2 phenotype in CD11b^+^ cells of spleen and inguinal lymph nodes. *Arg1* is an M2 marker and its expression was increased in spleen CD11b^+^ cells of *Shb* knockout mice with tumors compared with non-tumor bearing mice (Figure 8A). Expression of the M1 markers *Tnfa* was, on the other hand, decreased in spleens of tumor-bearing knockout mice when compared with spleens of tumor-bearing controls whereas expression of the M1 marker *Nos2* was unchanged. In inguinal lymph nodes of tumor bearing mice (control lymph nodes were too small for cell and RNA isolation), CD11b^+^ cells of *Shb* knockout mice expressed more *Arg1* whereas no difference in *Tnfa* expression could be observed (Figure 8B). Collectively, our results illustrate that CD11b^+^ cells of *Shb* knockout mice have become polarized towards a more M2-like phenotype.

## DISCUSSION

We presently describe vascular and immune cell responses to 4T1 breast carcinomas with particular reference to their dependence on the host *Shb* gene. SHB has previously been described in contexts of tumor angiogenesis [26, 32], vascular leakage [26, 27], myeloid infiltration [39] and T cell responses [30] and we thus considered it appropriate to study the effects of *Shb-*deficiency in the 4T1 breast carcinoma model.

The overall message is that 4T1 tumors hijack the host immune system in order to promote their expansion. The two main effects observed were global expansion of CD11b^+^ cells and a reduction in CD4 positive cells concomitant with a relative increase of IL-35-producing Treg cells. The relevance of an increase of Treg cells is further reinforced by the simultaneously occurring increase of plasma IL-35 in tumor-bearing wild type mice. Such an effect is anticipated to diminish a T cell response against tumor cells whereas the increase of CD11b^+^ cells is expected to produce tumor associated macrophages (TAM) that support tumor growth as stromal cells promoting tumor angiogenesis or exerting other supportive functions [6]. These data are in line with previous studies since it has been reported that IL-35 blocks the proliferation of T cells to dampen the inflammatory response [16, 17]. Furthermore, IL-35 increases the proliferation of M2 macrophages [40]. Moreover, myeloid cells/macrophages were readily detectable in tumors whereas the presence of CD4^+^ cells was less apparent.

In addition to the general effects of 4T1 tumors on responses of the immune system of wild type mice, certain effects specific to absence of the *Shb* gene were noted. These were an increased proportion of CD4/FoxP3/IL-35 positive cells in blood, a decreased proportion of CD11b/CD8 positive cells in lymph nodes and bone marrow and a decreased proportion of CD11c/CD8 positive cells in lymph nodes. Finally, CD11b^+^ cells of spleen and local lymph nodes showed increased expression of the M2 tumor supporting macrophage marker arginase 1 (*Arg1)* in *Shb* knockout mice. All these effects are expected to exert tumor-promoting effects and since they occur globally in the mouse they are in concert excellent candidates for the increased rate of tumor metastasis observed.

Tumor growth in itself was not significantly different between wild type or knockout mice. Although the vasculature showed differences between the genotypes suggesting that different angiogenic cues were operating, *Shb* knockout angiogenesis was apparently sufficient to support tumor expansion. Likewise was immune cell infiltration similar or identical, further in line with the similarity in tumor expansion. Finally, there were no differences in vascular leakage or pericyte coverage, reducing the likelihood that the difference in metastasis stems from an effect related to “leaky” vessels. Thus, the combined data lend further support to the hypothesis that the differences in immune responses detected in lymphoid organs outside the tumor itself are the most plausible explanations to the increased rate of metastasis. This implies that the tumor may cause immune cell alterations at sites distant from the tumor itself in a fashion dependent of the host’s genetic setup. Such an effect could in part occur via an arrest in the differentiation of bone marrow derived CD11b^+^ myeloid cells by release of tumor-derived factors. These immature myeloid cells could migrate to peripheral lymphoid organs, to the primary tumor to contribute to the formation of the tumor microenvironment, and could even be recruited to distant organs for the formation of an immunosuppressive pre-metastatic niche [41]. In agreement with the presence of metastases in sentinel lymph nodes in breast cancer patients, tumor cells can also be detected in lymph nodes in the 4T1 experimental mouse models [42]. Therefore, the enlarged inguinal lymph nodes in our study are potentially a pre-metastatic niche or a site of early metastasis.

VEGFA-dependent angiogenesis has been shown to play a role for 4T1 tumor growth and metastasis [43] although the effects of VEGFR2 inhibition were modest. The vascular plexa shown in Figure 2 are characteristic of VEGFA-dependent angiogenesis [44, 45] and their reduction in knockout tumors is in agreement with reduced VEGFA-dependent angiogenesis occurring under these conditions. This conforms with what was previously observed in RIP-Tag2 insulinomas [32], in which VEGFA-dependent angiogenesis was reduced by absence of the *Shb* gene but compensated for by other mechanisms, such as “inflammation” [21]. Upon VEGFR2-blocking treatment of 4T1 tumors, an increase in *Arg1* gene expression was noted and inhibition of this enzyme reduced metastasis, implicating arginase 1 in metastasis occurring in response to VEGFA inhibition [43]. The activation of immunosuppressive pre-metastatic niche cells in tumor-bearing conditions results in up-regulated expression of immune suppressive factors such as arginase 1 (encoded by *Arg1*), inducible nitric oxide synthase and Treg cells that inhibit effector T cell function [46].

It is curious that the tumor may influence both innate and adaptive immunity in multiple fashions that all in concert appear to promote tumor expansion. Furthermore, effects are exerted in the lymphoid system outside the tumor and these seem to play a role for the tumor’s ability to metastasize. Finally, the genetic composition (*i.e. Shb* knockout in present study) plays a role for the ability of the tumor to influence the immune response. Such a multi-facetted scenario is likely to play a role for the success of immune therapy. In all, the data suggest that multiple simultaneous approaches intervening with the immune system are probably required for a broader application of this therapy in the treatment of carcinoma.

## MATERIALS AND METHODS

### Animals

Eight- to 12-week-old female Shb+/+ or Shb-/- Balb/c mice were used in all experiments. All animal experiments were approved by the local animal ethics committee at the Uppsala county court.

### 4T1 primary tumor growth and spontaneous lung metastasis

The Balb/c mouse breast cancer cell line 4T1 was maintained in RPMI 1640 (Sigma-Aldrich) supplemented with 10% of fetal bovine serum (FBS) (Sigma-Aldrich). Cells were routinely maintained as subconfluent monolayers with splitting every 3 days and not kept in culture for more than 3 passages.

Balb/c Shb+/+ or Shb-/- female mice were inoculated in the fourth mammary fat pad with 10^6^ 4T1 cells in 50 μ l PBS. The tumor volume was measured with a caliper (calculated by the ellipsoid formula: volume = length × width^2^ × 0.52) every 2 or 3 days from day 11 after inoculation. At day 24-day 25 after inoculation, tumors were collected, weighed and snap-frozen when the tumor reached a size of about 500-600 mm^3^. All animals survived until the experimental end point at which the organs were collected for further analysis. In some experiments, primary tumors were resected at that time point. The mice were then kept for another an additional 8-14 days (until signs of disease appeared) to allow for scoring of lung metastases under extended conditions. To observe microsphere extravasation in 4T1 tumors, 100 μl of 30-nm fluorescent microspheres (G25, Thermo Scientific) was administered by tail vein injection ten minutes before collecting tumors. To score for metastatic spread, lung was inflated with OCT (Thermo Scientific) via trachea and snap-frozen for subsequent staining.

### Immunofluorescent staining

Excised tumors were sectioned, stained with CD31 (BD Pharmingen), Desmin (Abcam), VE-Cadherin (R&D systems), CD4 (Biolegend), CD8 (eBioscience), and CD68 (AbD Serotec) and imaged for 5 pictures / tumor using a Nikon fluorescence microscope. Supplementary Figure 9 shows CD31/Hoechst and Hematoxylin and eosin (HE) staining of consecutive sections demonstrating the presence of vascular plexa located both peripherally in the surrounding tissue and within the tumors. Lungs were sectioned, stained with Anti-Cytokeratin 19 antibody (Abcam) and HE to confirm the metastases. Quantification of vessel parameters was done using ImageJ (National Institutes of Health) in a blinded fashion.

### Isolation of CD11b cells from spleen and lymph nodes by magnetic cell sorting

Minced spleens and lymph nodes were passed through a 40μm strainer to get a single-cell suspension as described previously [16, 47]. After lysis of red blood cells, the cell suspension was incubated with CD11b microbeads mouse / human (130-049-601, Miltenyi Biotec) for 15 minutes at 4°C. CD11b-positive cells were eluted from the beads using MACS^®^ separation columns (130-042-201, Miltenyi Biotec) according to the manufacturer’s instructions and collected for subsequent RNA extraction.

### RNA extraction, quantitative PCR

Total RNA from 4T1 tumors or from magnetic cell sorting (MACS®)-isolated CD11b^+^ cells from spleens and lymph nodes was extracted using the Qiagen RNeasy kit following the manufacturer’s instructions. One-step quantitative real-time RT-PCR was performed with QuantiTect™ SYBR®Green RT-PCR-kit (204243, Qiagen) on a LightCycler™ real-time PCR machine (lightcycler 2.0; Roche). Cycle threshold (Ct) values were determined with the LightCycler Software. RNA expression was normalized by subtracting the corresponding β-actin Ct-value and relative values were calculated by the formula 2^(median of all ΔCt-ΔCt)^.

### Flow cytometry

Tumors were cut into small pieces and digested in 5 ml of Hanks’ solution (Sigma-Aldrich) including 1 mg/ml Collagenase A (C0130, Sigma) and 2mg/ml Dispase (D4693, Sigma) for 30-45 min at 37°C. The tumor suspension then was filtered through a 70 μm cell strainer. Alternatively, cell suspensions were prepared from excised thymi, spleens and inguinal lymph nodes as previously described [47]. Blood was drawn from hearts at the time of sacrifice and iliac, femur and tibia bones were dissected, crushed and cells were collected to obtain bone marrow cells. Red blood cells were lysed prior to staining.

Approximately 2-4 × 10^6^ cells were stained for flow cytometry analysis. The cells were first stained with cell surface antigens for 30 min on ice and then washed with flow cytometry staining buffer (eBioscience, San Diego, CA, USA). In order to stain for intracellular markers the cells were permeabilized and fixed by using Permeabilization-fixation buffer (eBioscience). After washing, cells were stained with intracellular markers for 60 min on ice. The cells were not stimulated prior to cytokine staining since that may cause artifacts upon Foxp3 staining [48]. Antibodies from eBioscience were as follows: anti-CD4 and anti-Foxp3. The following antibodies were from Biolegend, San Diego, USA: anti-B220, anti-CD1d, anti-CD11b, anti-CD11c, anti-Ly6G and anti-PDCA-1. The anti-CD8 and CD19 were from BD and anti-Ebi3 and anti-IL-12p35 were from R&D, Minneapolis, MN, USA.

The stained cells were analyzed on a BD LSRII Flow Cytometry (BD) at the core facility (BioVis) Uppsala University, Uppsala, Sweden. The data were analyzed with FlowJo software (FlowJo, LLC, Ashland, OR, USA). Gating strategies were made using single stained and unstained controls as described earlier [47].

### IL-35 ELISA

The circulating concentration of IL-35 in plasma was determined by using an IL-35 ELISA kit from Wuhan USCN Business Co, Ltd., China.

### Statistical analysis

Statistical analyses were performed using GraphPad Prism 7.0 Software (GraphPad Software, Inc.). All values are presented as means ± SD. Paired (tumor size) or unpaired (vessel parameters) two tailed Student *t*-test was used for comparison between two groups. P < 0.05 was considered as statistically significant. For the FACS data, Fisher’s LSD test was used when comparing wild type tumor with wild type non-tumor or KO tumor.

## SUPPLEMENTARY MATERIALS FIGURES



## Abbreviations

SHB: Src homology 2 domain protein B
IL: interleukin
CD: cluster of differentiation
EMT: epithelial-mesodermal transition
T_reg_: regulatory T cells
Th: T helper cells
VEGF: vascular-endothelial growth factor
RIP-Tag2: rat insulin promoter SV40 large T antigen
RT-qPCR: real-time reverse-transcriptase polymerase chain reaction
FACS: fluorescence activated cell sorting
FoxP3: forkhead box P3
M: macrophage
TAM: tumor associated macrophages
VE: vascular endothelial
HE: hematoxylin and eosin
