# Supplementary material for: Pro-tumoral immune cell alterations in wild type and Shb-deficient mice in response to 4T1 breast carcinomas
Source: Oncotarget. 2018 Apr 10;9(27):18720–33. doi: 10.18632/oncotarget.24643 (PMC5922350; doi:10.18632/oncotarget.24643)
Supplement: Supplementary file 1 [file oncotarget-09-18720-s001.pdf]

Pro-tumoral immune cell alterations in wild type and *Shb*-deficient mice in response to 4T1 breast carcinomas

SUPPLEMENTARY MATERIALS

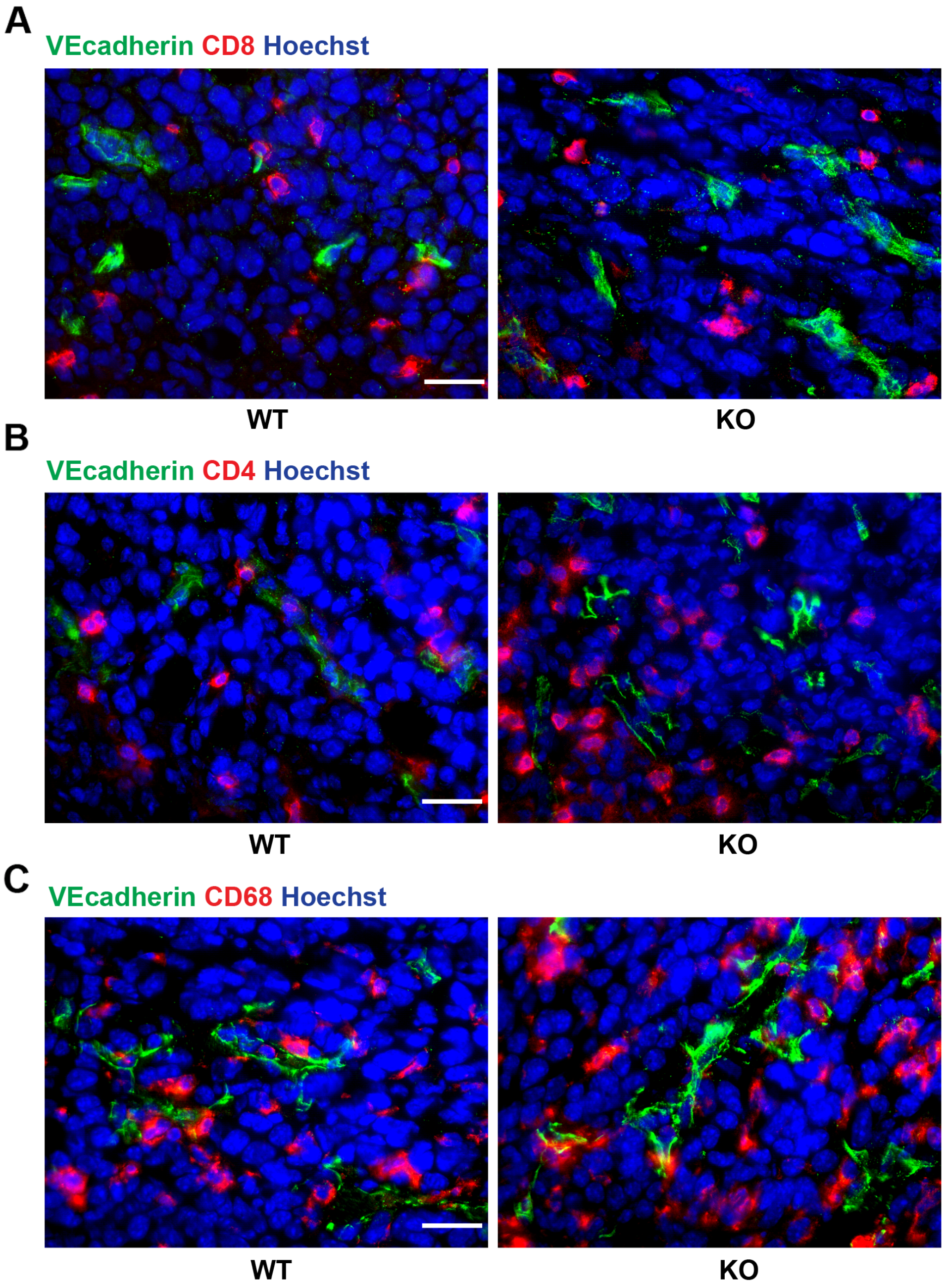

Supplementary Figure 1: Tumor staining for CD8 **(A)**, CD4 **(B)** and CD68 **(C)** after growth in wild type and *Shb* knockout mice. Parts of tumors were frozen in liquid nitrogen at the time of sacrifice and subsequently cryosectioned for immunfluorescence staining (secondary antibody donkey anti-rat alexa594. The sections were counterstained for VE-cadherin (secondary antibody donkey anti-goat alexa488) and Hoechst. Scale bar 20 µm.

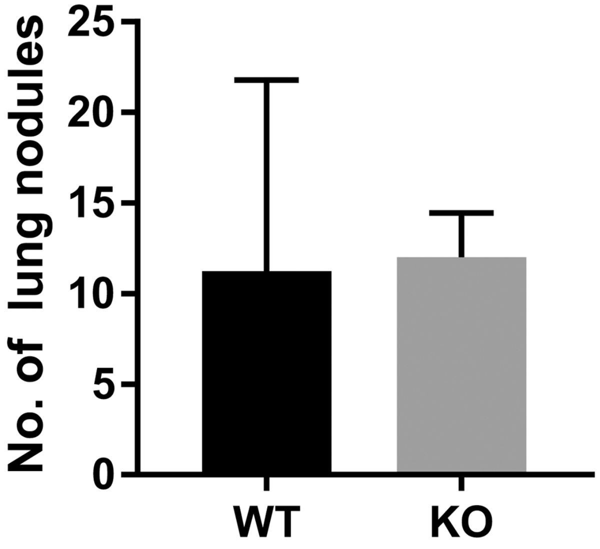

Supplementary Figure 2: Lung seeding of tail vein injected 4T1 cells. One half million 4T1 cells were tail vein injected and the mice were sacrificed 22-24 days later. Lung metastases were counted. Means ± SD for 4 mice of each genotype are given.

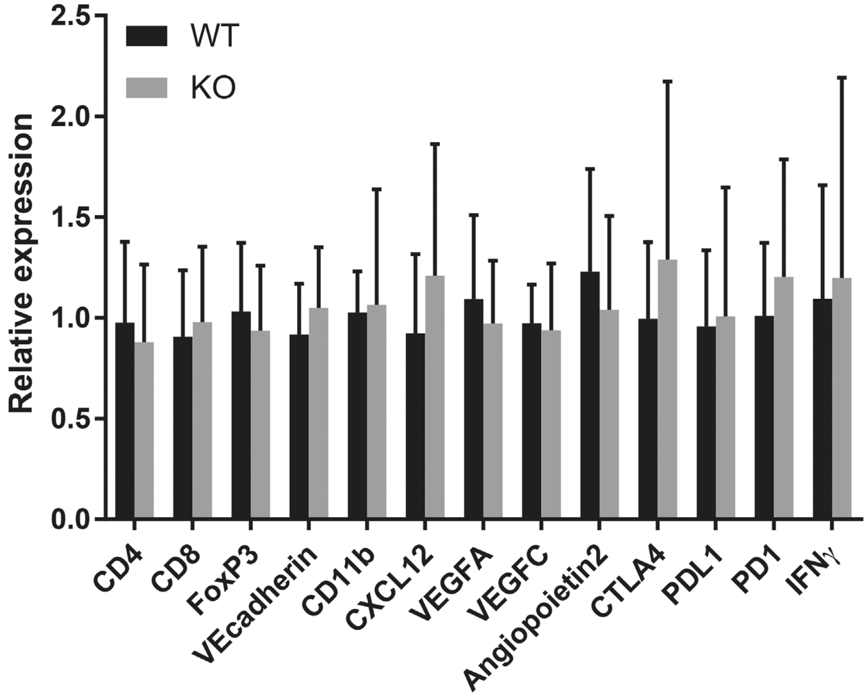

Supplementary Figure 3: Tumor gene expression by real-time RT-PCR (qPCR). Tumor RNA was isolated at the time of sacrifice. Relative values are given. Means ± SD are shown. N= 7 mice each genotype.

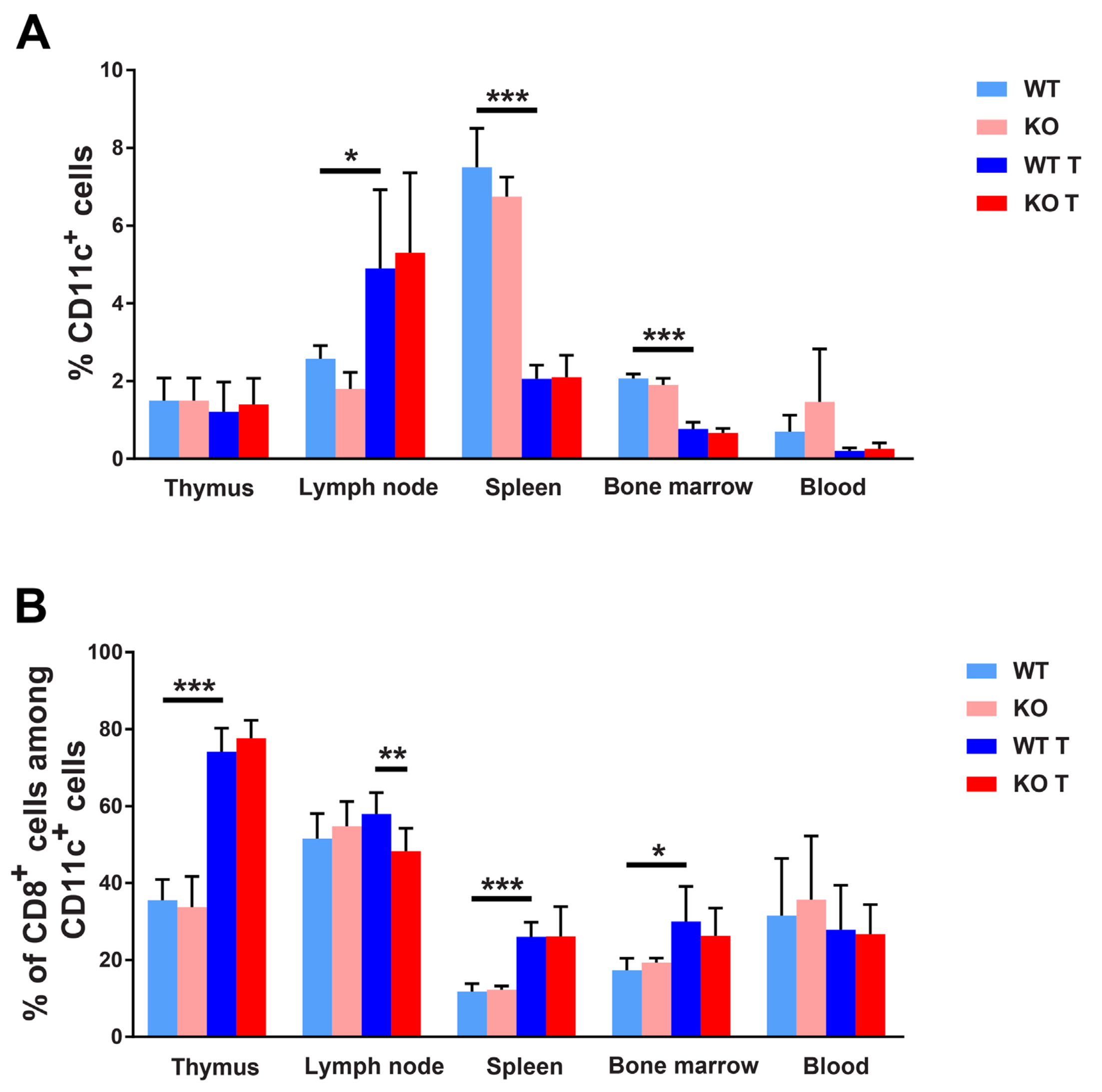

Supplementary Figure 4: FACS analysis of CD11c and CD8/CD11c double positive cells in lymphoid organs. (A) CD11c positive cells in different lymphoid organs. **(B)** CD8/CD11c positive cells are given in percent of CD11c positive cells. Lymphoid organs were collected and single cells prepared at the time of sacrifice of age-matched non-tumoral mice and tumor bearing mice. Means ± SD are given. *, ** and *** indicate p<=0.05, 0.01 and 0.001, respectively when compared as indicated with wild type tumor mice using Fisher's LSD test. N= 4 for non-tumor and 7-10 for tumor.

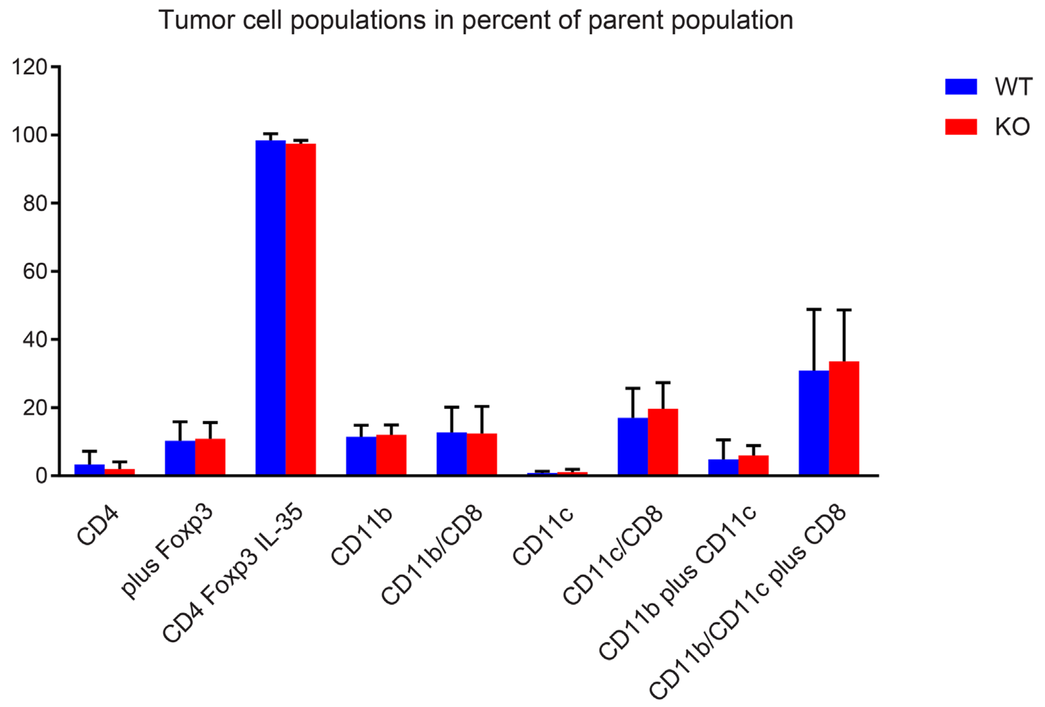

Supplementary Figure 5: Tumor FACS analysis for different markers of innate or acquired immunity. Single cells were prepared from tumor at the time of sacrifice and subjected to staining and FACS analysis for CD4, CD8, FoxP3, IL-35, CD11b and CD11c. Values are percent of parent population. Means ± SD are given for 7 observations.

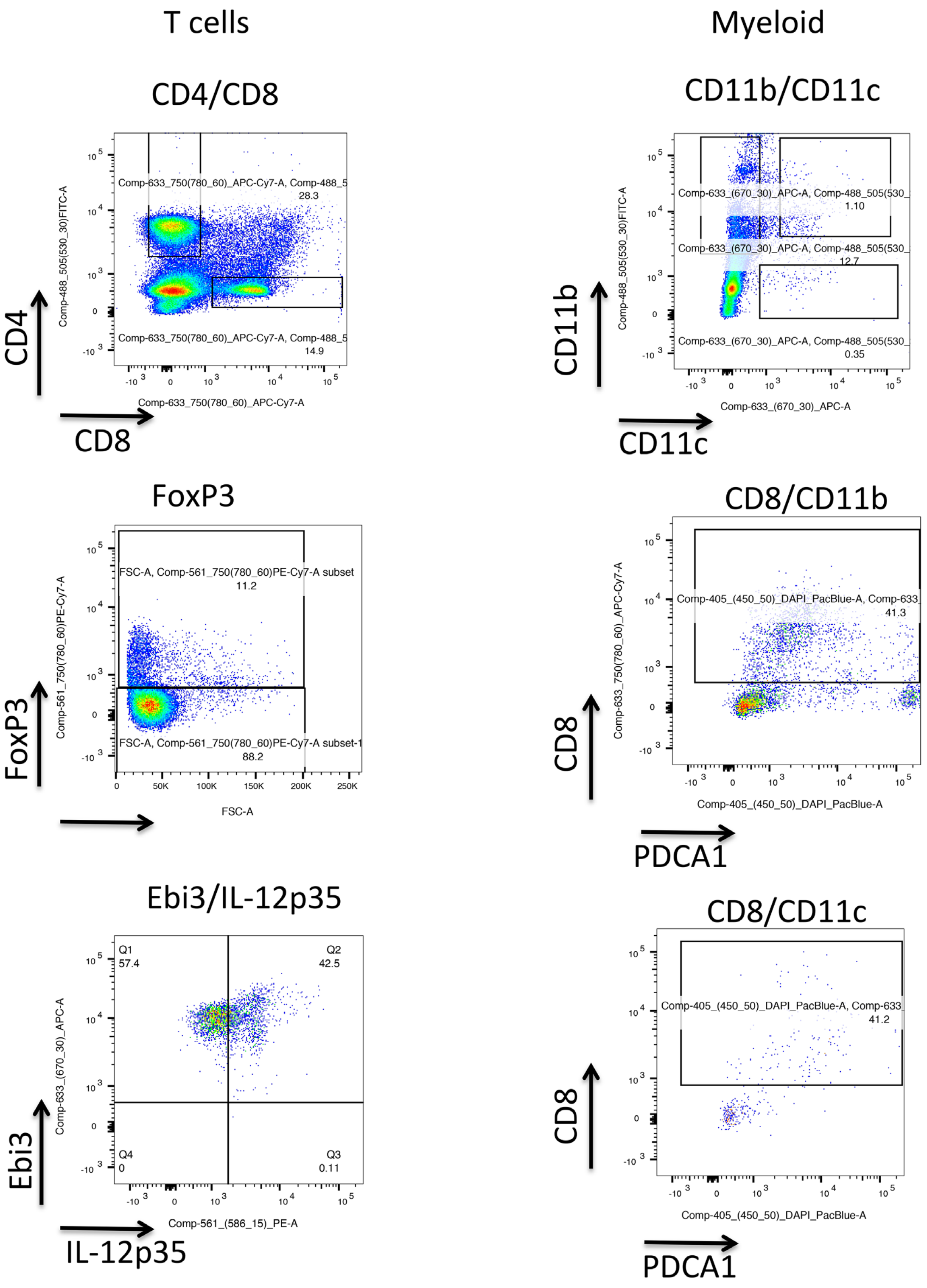

Supplementary Figure 6: Representative FACS plots showing the gating strategy. Lower panels show plots of gated population in the corresponding upper panels. PDCA-1 staining (plasmacytoid dendritic cell antigen-1) was also performed but the data not presented in the text since no differences could be detected.

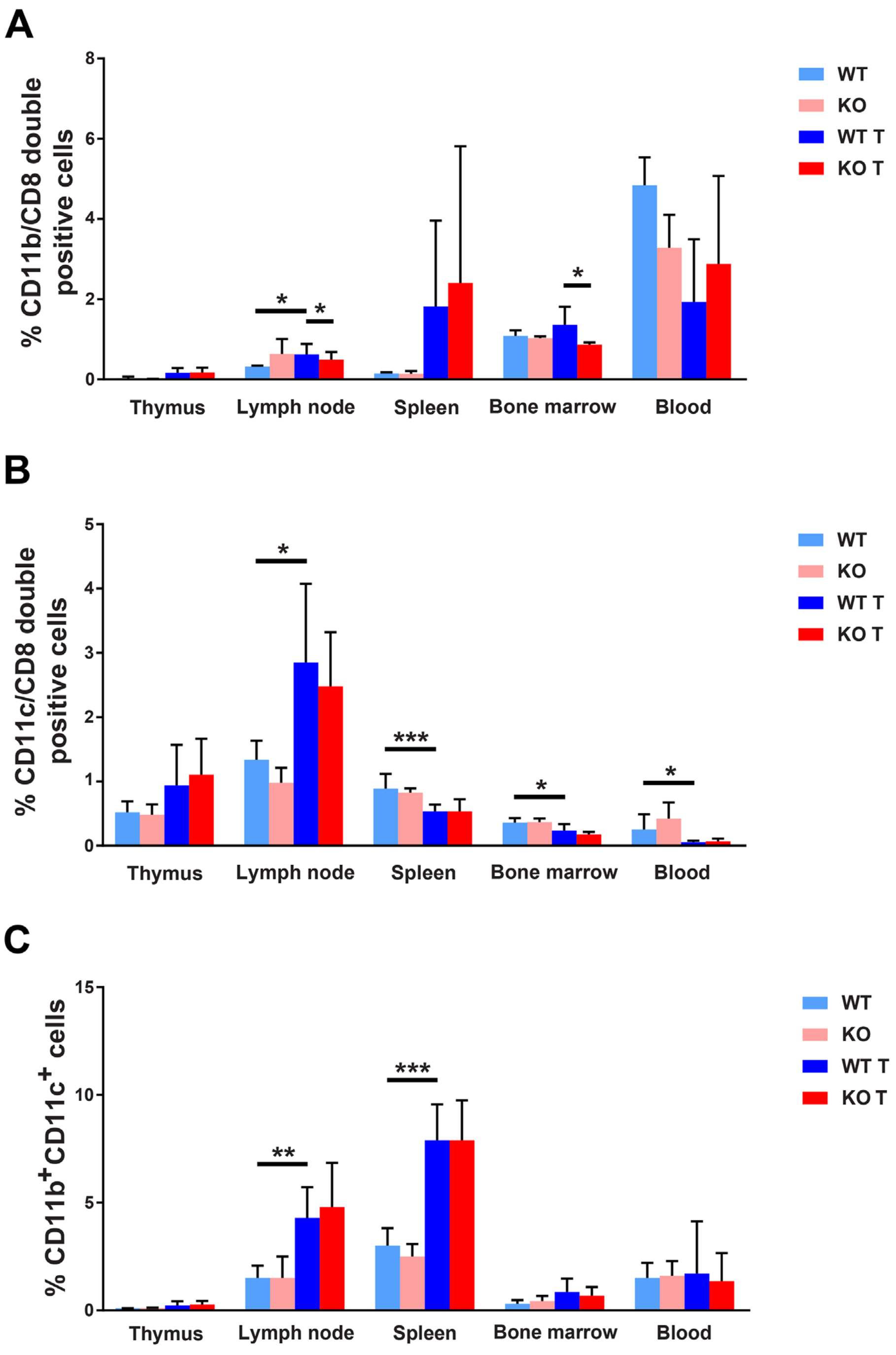

Supplementary Figure 7: CD8/CD11b **(A)** and CD8/CD11c **(B)** double positive cells in percent of total viable cell population. Numbers from Figure 5 and Supplementary Figure 4 were multiplied and divided by 100 to obtain percentages double positive cells of total cell population. CD11b/CD11c double positive cells are shown in **(C)**. Means ± SD are given. *, ** and *** indicate $p<0.05$, 0.01 and 0.001, respectively, when compared with wild type tumor control by Fisher's LSD test.

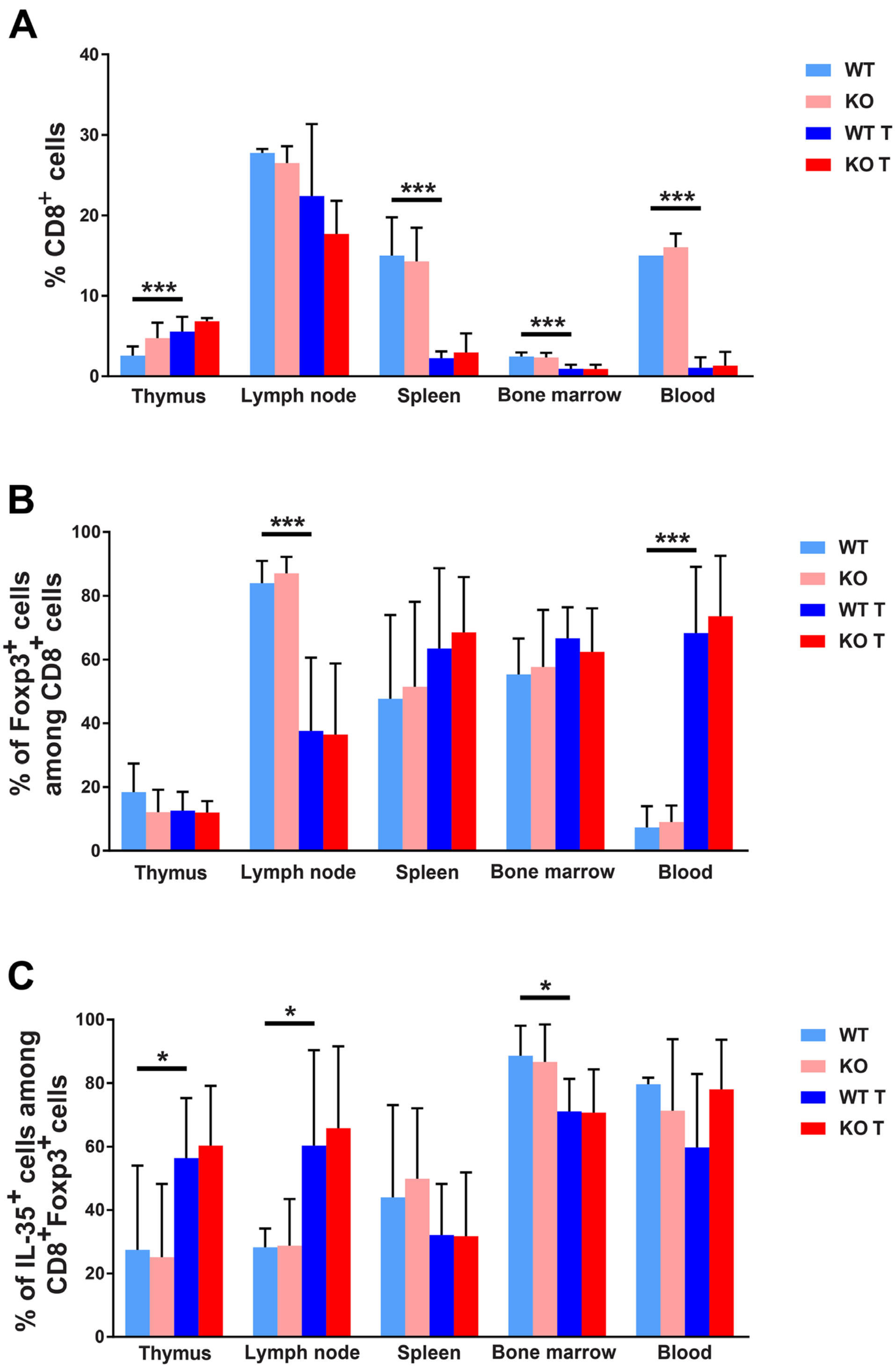

Supplementary Figure 8: FACS staining for CD8+ Tregs. **(A)** CD8 positive cells (percent) in different lymphoid organs. **(B)** Foxp3 positive cells as percentage of CD8+ cells. **(C)** IL-35 positive cells in percent of FoxP3/CD8 positive cells. Means ± SD are given. * and *** indicate p<=0.05 and 0.001, respectively when compared as indicated with wild type tumor mice using Fisher's LSD test. N= 4 for non-tumor and 7-10 for tumor mice

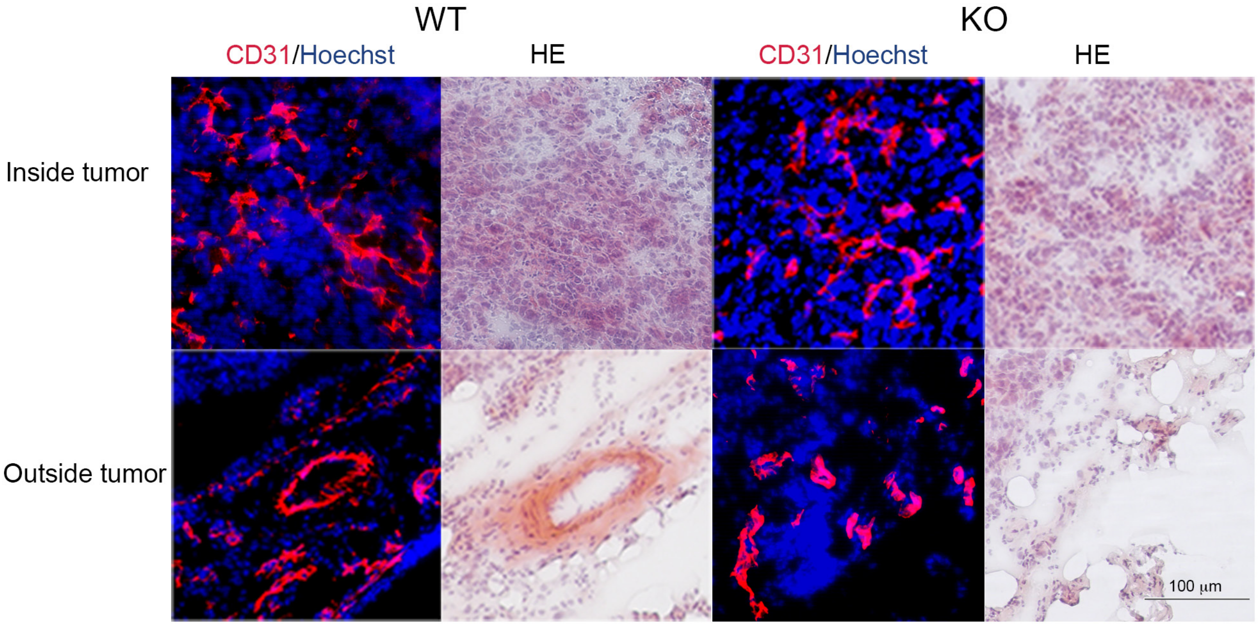

Supplementary Figure 9: Vascular plexa in the surrounding tissue and within the primary tumors. Wild type and *Shb* knockout primary tumors were stained for CD31/Hoechst and HE in consecutive sections. Pictures show plexa located inside the tumor and in the peripherally surrounding tissue.
